# Effect of the Strain Rate on Damage in Filled EPDM during Single and Cyclic Loadings

**DOI:** 10.3390/polym12123021

**Published:** 2020-12-17

**Authors:** Nicolas Candau, Oguzhan Oguz, Edith Peuvrel-Disdier, Jean-Luc Bouvard, María Lluïsa Maspoch, Guillaume Corvec, Christophe Pradille, Noëlle Billon

**Affiliations:** 1 Centre Català del Plàstic (CCP)—Universitat Politècnica de Catalunya Barcelona Tech (EEBE-UPC), Av. D’Eduard Maristany, 16, 08019 Barcelona, Spain; maria.lluisa.maspoch@upc.edu; 2 Faculty of Engineering and Natural Sciences, Materials Science and Nano Engineering, Sabanci University, Orhanli, Tuzla, Istanbul 34956, Turkey; oguzhanoguz@alumni.sabanciuniv.edu; 3 Sabanci University Integrated Manufacturing Technologies Research and Application Center & Composite Technologies Center of Excellence, Teknopark Istanbul, Pendik, Istanbul 34906, Turkey; 4 Mines ParisTech, CEMEF—Centre de Mise en Forme des Matériaux, UMR CNRS 7635, PSL Research University, CS 10207, 06904 Sophia-Antipolis, France; edith.peuvrel-disdier@mines-paristech.fr (E.P.-D.); Jean-Luc.Bouvard@mines-paristech.fr (J.-L.B.); guillaume.corvec@mines-paristech.fr (G.C.); noelle.billon@mines-paristech.fr (N.B.); 5 Mat-xper, 06560 Valbonne, France; christophe.pradille@mat-xper.com

**Keywords:** digital image correlation, damage, infra-red thermography, self-heating, EPDM

## Abstract

The effect of the strain rate on damage in carbon black filled Ethylene Propylene Diene Monomer rubber (EPDM)stretched during single and multiple uniaxial loading is investigated. This has been performed by analyzing the stress–strain response, the evolution of damage by Digital Image Correlation (DIC), the associated dissipative heat source by InfraRed thermography (IR), and the chains network damage by swelling. The strain rates were selected to cover the transition from quasi-static to medium strain rate conditions. In single loading conditions, the increase of the strain rate yields in a preferential damage of the filler network while the rubber network is preserved. Such damage is accompanied by a stress softening and an adiabatic heat source rise. Conversely, increasing the strain rate in cyclic loading conditions yields in a filler network accommodation and a high self-heating whose combined effect is proposed as a possible cause of the ability of filled EPDM to limit damage by reducing cavities opening during loading, and favoring cavities closing upon unloading.

## 1. Introduction

The fracture behavior of rubber materials during service is widely affected by its thermomechanical history, in turn governed by the loading conditions. Strain rate is a crucial parameter that requires special attention, as it influences the kinetics of nucleation and growth of cavities, and by inference impacts the ultimate mechanical properties of rubber. The effect of the strain rate on damage is of significance, especially when elastomers undergo severe loading conditions, like high frequency cyclic loading [1]. Such conditions are met in many industrial applications such as rubber fatigue in pneumatic tires [2], mechanical devulcanization of wastes rubber [3,4], cyclic compression of cellular elastomers [5], and dynamic loading of rubber springs [6]. Moreover, the recent progress in automation of the testing methods and limited computational time due to algorithm improvements allows us to investigate damage mechanisms at larger strain rate range [7], expanding the investigation of damage behavior on a wider number of applications [8].

The impact of strain rate on the macroscopic mechanical behavior of filled elastomers has been extensively studied in the literature from experimental and theoretical points of view [9,10]. It is admitted that the equivalence time-temperature is applicable to the stress softening in filled rubbers to describe the viscoelastic behavior [9]. The higher the strain rate, the lower the temperature, the higher the stress softening. Hysteresis loss induced by pre-stretching of filled rubber is significantly increased at high strain, implying the manifestation of dissipative mechanisms [10]. Among these dissipative mechanisms, the increased viscoelasticity with strain rate may be due to the increased amount of trapped entanglements and chains slippage (molecular friction) in the bulk rubber and at filler interface.

Concomitantly, the increase of strain rate may favor another dissipative mechanism, namely damage. Such damage may occur in the rubber network due to increased contribution of chains scission [11] and sulfur bond breakage, and breakage of ZnO [12] and ZnS [13] aggregates in case of sulfur-vulcanized rubbers. Such damage may also occur in the filler network involving filler-rubber rupture or breakage of inter-aggregate bonds [14]. Hence, in addition to the macroscopic mechanical behavior, it is of great importance to investigate the effect of the strain rate on associated damage mechanisms in filled elastomers, as they are at the origin of rubber macroscopic failure. Damage being associated with void creation, it generates a raise of volumetric strain easily accessible by Digital Image Correlation (DIC) [15,16,17,18]. Investigations on damage in elastomers are commonly conducted in quasi-static conditions, i.e., from 10^−4^ s^−1^ to 10^−1^ s^−1^, as defined in Ref. [7]. In these conditions, the observed damage was found weakly dependent on strain rate [19]. In addition, no associated dissipative heat was expected.

As the strain rate further increases, the transition from isothermal to adiabatic conditions attests for the appearance of thermal dissipation. The temperature rises because mechanical work is converted into heat, which, in the absence of phase transition, is generally governed by viscoelasticity and damage occurring at large strain. By analyzing the heat sources based on infrared thermography analysis, Le Cam et al. showed that the damage in filled rubbers can be separated from other dissipative mechanisms [20,21]. In turn, the increased self-heating due to high frequency mechanical cycles, namely heat build-up, is supposed to have a crucial influence on the fatigue damage in filled elastomers. As a study case, filled natural rubber cyclically stretched at high frequency undergoes self-heating, decreasing its ability to crystallize [22] and limiting its fatigue lifetime [23], that is finally governed by the development of cavitation. Following the Risitano method [24], by observing the evolution of fatigue damage, self-heating was found to be correlated with the fatigue lifetime in both crystallizing and non-crystallizing filled rubber [25].

It has been pointed out that the development of adiabatic models accounting for self-heating is crucial in order to properly describe the damage behavior of filled elastomers, especially when combining high strain rate and cyclic loading conditions [26]. However, to the authors’ knowledge, there is a lack of systematic experimental study of damage over a strain rate range covering the transition from quasi-static to medium strain rate conditions, exploring the possible coupling between the occurrence of damage, stress relaxation, and heat dissipation.

In this paper, the effect of the strain rate on damage in carbon black filled Ethylene Propylene Diene Monomer rubber (EPDM) stretched during single and multiple uniaxial loading is investigated. This has been performed by analyzing the stress–strain response, the voiding fraction by Digital Image Correlation (DIC), the associated dissipative heat source by infrared thermography (IR), and the chains network damage by swelling. The strain rates are selected so that they cover the transition from quasi-static to medium strain rate conditions with the aim of further understanding the thermomechanical coupling associated with damage in rubber materials. In a first part, it is shown that filler network damage yields in heat source rise and favors stress softening during single tensile tests. In a second part, the combined effect of filler network accommodation and high self-heating due to the accumulation of high strain cycles is also highlighted as a possible cause of the ability of highly filled EPDM to limit the development of cavitation by reducing the amplitude of voids opening/closing.

## 2. Materials and Methods

### 2.1. Materials Composition and Processing

The materials are extended oil carbon black filled Ethylene Propylene Diene Monomer rubber EPDM Keltan 5470 (SACRED Group, Saint-Lubin-des-Joncherets, France) obtained by sulfur vulcanization of the gum. They contain 70% in mass of Ethylene and 4.6% of ethylidene norbornene (ENB). They all contain 4 phr of calcium oxide, 5 phr of zinc oxide, 1 phr of stearin, and 2 phr of Polyethylene Glycol (PEG 4000) as plasticizer. The vulcanization components are 1.2 phr of sulfur (75%), 1 phr of mercaptobenzothiazole (MBT 75%), 0.8 phr of mercaptobenzothiazole disulfide (MBTS 75%), 1.2 phr of N-cyclohexyl-2-benzothiazolesulfenamide (CBS 75%), and 1.5 phr of zinc dialkyl dithiophosphate (ZDTP 70%). All materials contain 0, 40, or 80 phr (phr means per 100 g of rubber) of carbon black (N550), whose BET surface area (NSA) is 40 m^2^·g^−1^ and the external surface area (STSA) is 39 m^2^·g^−1^. The composition of the materials is summarized in Table 1. From the knowledge of the density (g.cm^−3^) of each component provided in this table, the weight fraction of carbon black particles corresponding to 40 phr and 80 phr are found equal to 25 wt.% and 40 wt.%, respectively. An internal mixer (Brabender^®^ GmbH & Co. KG, Germany) composed of co-rotating twin screws is used for the rubber compounding. The volume introduced in the chamber was 40 cm^3^ corresponding to a filling factor of 0.9. The mixing rate is fixed at 40 rpm and the temperature at 60 °C. The rubber was blended first, and after five minutes the carbon black particles were added, and then the processing continued for five more minutes. The vulcanizing system was subsequently added in an open roll mill. Sample sheets are produced by curing in a hot press at 170 °C. By using a Rubber Process Analyzer (RPA), the curing time t_100%_ = 15 min, a time corresponding to the moment when the torque reaches its maximum value. Dumbbell-shaped samples, with a 15 mm gauge length (*l*_0_), 6 mm thickness (*e*_0_), and 10 mm width (*L*_0_), are then prepared for tensile testing.

### 2.2. Thermomechanical Set-up by Infrared Spectroscopy (IR) and Digital Image Correlation (DIC)

An electromechanical tensile test machine (INSTRON 5960 Series Universal Testing Systems, Norwood, MA, US) is used to perform mechanical tests (both single loading and cyclic loading) at room temperature. The nominal tensile strain rate of 10^−2^·s^−1^ (0.15 mm·s^−1^), 10^−1^·s^−1^ (1.5 mm·s^−1^), and 1·s^−1^ (15 mm·s^−1^) are applied for these tests. Each test was performed five times to ensure the reproducibility. A four-camera setup consisting of two-pairs systems is used to record the displacement fields on both front and side faces of the sample. Both systems are respectively mounted with 25 mm and 50 mm (Schneider Kreuznach, Bad Kreusnash, Germany) objectives. The experimental set-up has been fully described in a previous work [27], and the main steps are reported here. The post-processing of a series of images recorded is performed using VIC-3D software package (VIC-3D Correlated solutions, Irmo, SC, USA). 3D DIC displacement data are converted into strain values. *λ*_1_ represents the stretching ratio in the tensile direction, whereas *λ*_2_ and *λ*_3_ correspond to the transversal stretching ratios in the directions of the sample width and thickness, respectively. The transversal and longitudinal strains are homogeneous in the center of the sample and significantly decrease close to the clamping zones. The strain fields in both front and side surfaces are determined from a region of interest (ROI) in the central part of the sample. Details on the choice of DIC parameters can be found in reference [27]. The true stress is defined as the ratio between the applied force F and the specimen cross-section:

(1) $$\;\sigma_{T}=\frac{F}{L_{0}e_{0}\lambda_{2}\lambda_{3}}$$

where *L*_0_ = 10 mm is the initial width and *e*_0_ = 6 mm the initial thickness. The volumetric strain ∆*V/V*_0_ is defined as:

(2) $$∆V/V_{0}(\lambda )=\lambda_{1}\lambda_{2}\lambda_{3}-1$$

Rubber samples are stretched at different maximum strains. The strain field in both front and side faces are obtained in the central part of the sample. Trace of self-heating generated in the sample is recorded on its surface during the deformation using FLIR SC5000 camera. The temperature field is analyzed on the front face of the specimen with ALTAIR software [28] in the same ROI than for DIC analysis.

### 2.3. Swelling

Each sample is immersed in cyclohexane for 72 h, and the solvent is replaced every 24 h. After 72 h, the swollen mass of each sample (𝑚_𝑠_) is measured. The samples are then placed in a vacuum oven at 70 °C for 6 h to ensure the complete removal of the solvent. The mass of the dry samples (𝑚_𝑑_) is then determined. The swelling ratio of the specimen (*Q*) was calculated following Ref. [29]. The network chain density is calculated from the swelling experiments and the Flory–Rehner equation [30]:

(3) $$\upsilon =\;\frac{ln\left(1-v_{2}\right)+v_{2}+\chi_{1}v_{2}{}^{2}}{V_{1}\left(-v_{2}{}^{\frac{1}{3}}+\frac{2}{f}v_{2}\right)}$$

where $v_{2}=1/Q$, *V*_1_ = 108 cm^3^·mol^−1^ is the molar volume of the solvent (cyclohexane), *χ*_1_ is the Flory–Huggins polymer solvent dimensionless interaction term (*χ*_1_ is equal to 0.353 for the EPDM–cyclohexane system). The ratio *2/f* is associated with the phantom model that assumes spatial fluctuation of crosslinks (non-affine) used for high deformation ratios. *f*, the crosslink functionality, is chosen equal to 4. For filled compounds, the Kraus correction [31] is used to account for the contribution of filler in swelling ratio. *Q_c_* is the swelling ratio of the rubber matrix defined as follows:

(4) $$Q_{c}=\frac{Q-\phi }{1-\phi }$$

where *ϕ* is the volume fraction of fillers. Krauss correction in Equation (4) assumes non-adhesion of the fillers to the rubbery matrix, suggesting damage in mechanically tested specimen may partly be ascribed to decohesion at filler–rubber interface.

## 3. Results and Discussion

### 3.1. Effect of the Strain Rate and Type of Loading on Chains Network Damage

In vulcanized filled rubber, chains are elastically active due to trapped entanglements, sulfur bonds, or bonds at filler interface. These elastically active chains are expected to experience some damage upon deformation via chains scission, breakage of chemical crosslinks, and breakage at filler interface. Due to the large distribution of network chains density in vulcanized rubber materials [32,33], strain induced damage in the rubber bulk, i.e., damage of the rubber network caused by chains scission and breakage of chemical crosslinks, is supposed to mostly occur in the bulk domains containing the shortest chains [29] and preferentially in the interfacial region between fillers and rubber molecules [11,34]. In the single tensile test, such rubber network damage may occur prematurely as compared to filler network damage [35].

**Figure 1:**
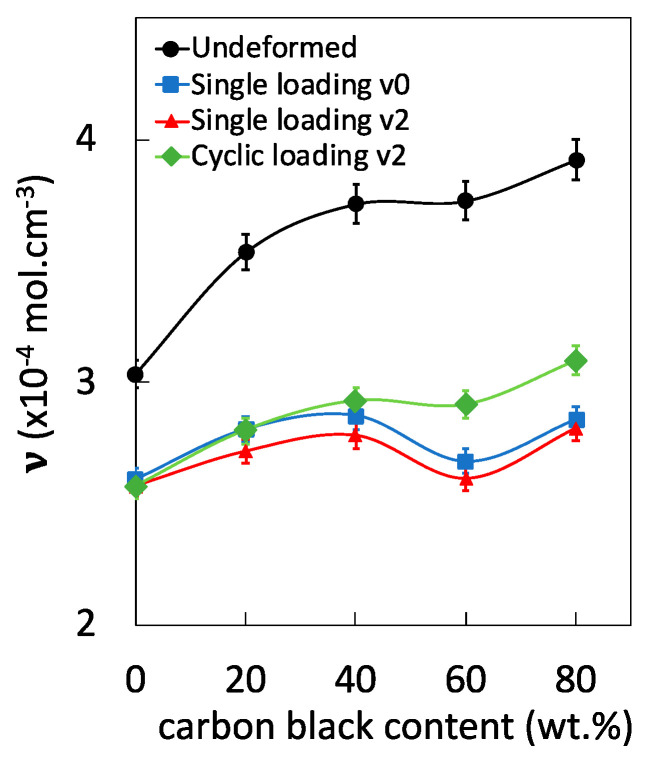
Network chain densities of EPDM with filler content of 0, 20, 40, 60, and 80 phr for untested (undeformed) specimens (ring symbols), for cyclically loaded specimens at the nominal strain rate 1 s^−1^ (diamond symbols), for single loaded specimens at the nominal strain rate 10^−2^ s^−1^ (square symbols), and for single loaded specimens at the nominal strain rate 1 s^−1^ (square symbols).

Here, the effect of the strain rate and of the type of loading on chains network damage in filled EPDM is investigated. In the literature, the macroscopic density of dry rubber specimens (unswollen) that have been tested mechanically and relaxed has been estimated. It was found to be independent of their previous mechanical history [36]. This may be partially ascribed to cavities closing upon unloading. Instead, here we aimed to perform post-swelling experiments (swelling on unloaded rubber specimen after mechanical tests) that expectedly allow us to re-open cavities, at least partially. This was done on mechanically tested EPDM containing various filler content of 0, 20, 40, 60, and 80 phr (Figure 1). For undeformed, unfilled EPDM-0 that did not experience mechanical loading, the network chains density has been calculated by using swelling ratio and the Flory-Rehner equation (see Section 2.3). The network chain density in undeformed filled rubbers has then been deduced from the normalized size in thermoporosimetry experiments, as detailed in a previous work [35]. Swelling on mechanically tested EPDM clearly shows an effect of the loading on chains network alteration, i.e., a decrease of the network chains density as compared to untested (undeformed) EPDM (Figure 1).

In addition, an increase of the strain rate slightly increases the level of chains network alteration of tested EPDM specimens. This is consistent with previous results from the literature [9]. The relative loss of network chain density by increasing the strain rate from 10^−2^ ·s^−1^ to 1 s^−1^ is found between 1% and 3%, which is weak as compared to the overall deformation effect (comparison between untested and tested specimens at the two tested strain rates). This suggests the strain rate effect involves damage of the filler network, as will be discussed in Section 3.2. Strikingly, the cyclic loading seems to be more favorable to the preservation of the chains network than monotonic loading (Figure 1), especially for filled EPDM with carbon black content above 40 phr. This suggests the damage of the rubber chains network to be limited in cyclic loading conditions, and the possible participation of the filler network to accommodate the deformation. The origin of such results will be discussed hereafter in the light of a thermomechanical analysis.

### 3.2. Effect of the Strain Rate on Damage during Single Loading

**Figure 2:**
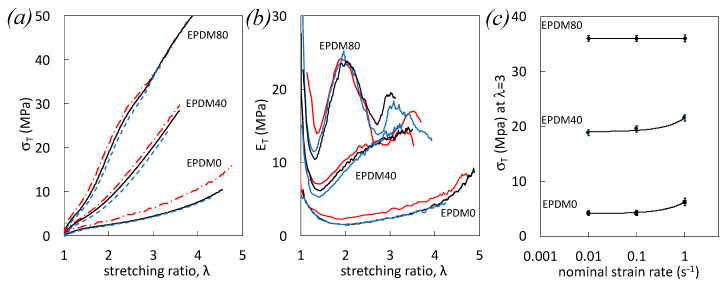
True stress (**a**), tangent modulus (**b**) versus strain for EPDM-0, EPDM-40, and EPDM-80 during single uniaxial loading at the nominal strain rates 10^−2^ s^−1^ (blue dotted line), 10^−1^ s^−1^ (black continuous line), and 1 s^−1^ (red dashed dotted line). (**c**) True stress at λ = 3 versus nominal strain rate for EPDM-0, EPDM-40, and EPDM-80.

**Figure 3:**
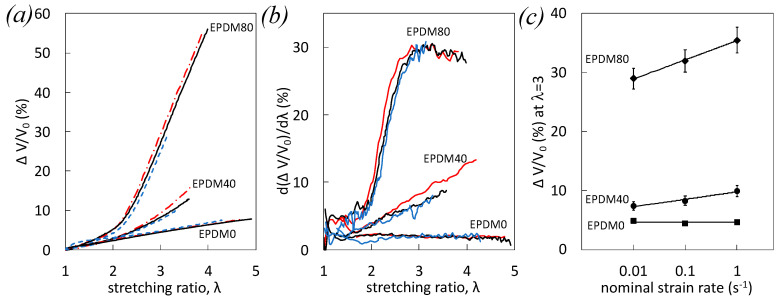
Volumetric strain (**a**) and rate of volumetric strain (**b**) versus strain for EPDM-0, EPDM-40, and EPDM-80 during single uniaxial loading at the nominal strain rates 10^−2^ s^−1^(blue dotted line), 10^−1^ s^−1^ (black continuous line), and 1 s^−1^ (red dashed dotted line). (**c**) Volumetric strain at λ = 3 versus nominal strain rate for EPDM-0, EPDM-40, and EPDM-80.

**Figure 4:**
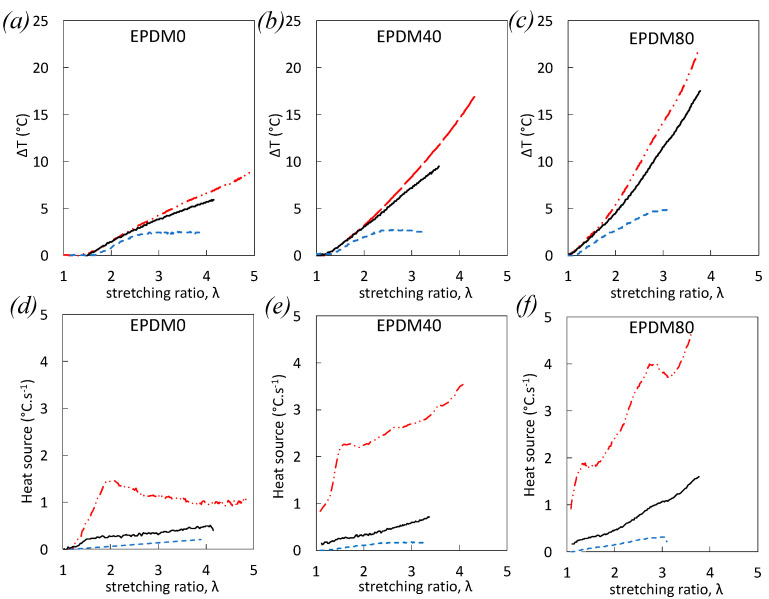
Self-heating versus strain for EPDM-0 (**a**), EPDM-40 (**b**), and EPDM-80 (**c**) during single uniaxial loading at the nominal strain rates 10^−2^ s^−1^(blue dotted line), 10^−1^ s^−1^ (black continuous line), and 1 s^−1^ (red dashed dotted line). Corresponding heat source versus strain for EPDM-0 (**d**), EPDM-40 (**e**), and EPDM-80 (**f**).

The mechanical behavior (Figure 2), damage (Figure 3), and heat sources (Figure 4) of filled EPDM subjected to single tensile tests are studied at various nominal strain rates distributed over two decades (from 10^−2^ s^−1^ to 1 s^−1^). The stress-strain curves of unfilled EPDM-0 show a higher deformation resistance with increased strain rate (Figure 2a). This is likely ascribed to increased viscoelasticity due to (i) higher number of elastically active chains through a higher number of trapped entanglements, and (ii) higher viscous resistance due to chain slippage (greater frictional resistance) [37,38]. At a given strain, the stress increases by increasing the filler content due to the reinforcing effect of fillers by hydrodynamic effect (strain amplification in presence of non-deformable particles), filler-filler, and filler-rubber interactions. By increasing the strain rate, viscoelasticity increases, for the same reason than as unfilled rubber, but additional effects may arise from higher frictional resistance due to chains slippage at the filler-rubber interface.

The strain and strain rate dependency of the tangent modulus, estimated as the derivative of the stress versus strain, is presented (Figure 2b). In unfilled EPDM-0 and filled EPDM-40, tangent modulus reveals a first decrease and increase stress upon loading, and a mechanical reinforcement with strain is clearly observed by increasing the strain rate (Figure 2b). Contrarily, EPDM-80 reaches a maximum tangent modulus at around λ = 2 before decreasing again and finally slightly increasing before failure. Such a trend is observed for the three studied strain rates, with a higher decrease of modulus before failure for the highest strain rate. This would suggest a predominance at large strain and high strain rates of damage that may participate to soften that material.

This peculiar stress softening is further studied by plotting the stress at large deformation (λ = 3) as a function of the strain rate (Figure 2c). First, increased stress with strain rate on unfilled EPDM-0 and filled EPDM-40 (Figure 2c) is in accordance with the above explanations. Surprisingly, the stress at high deformation (λ = 3 and above) in the highly filled EPDM-80 does not show strain rate dependence in the strain rate range studied (Figure 2c).

A direct measure of voids volume fraction can be assessed by the volumetric strain obtained by the four-camera DIC system presented in the experimental section. The voiding rate—tentatively describing the speed of voids formation into the materials upon loading—has been defined as the derivative of the volumetric strain as a function of the longitudinal strain (so-called “strain” or “stretching ratio”) and provides further information on the strain and strain rate dependence of voiding mechanisms.

Strain, strain rate, and filler dependence of volumetric strain (Figure 3a) and voiding rate (Figure 3b) are shown. As expected, at a given strain and strain rate, the volumetric strain as well as the voiding rate increase with filler content due to a higher level of damage at the filler-rubber and filler-filler interfaces. Volumetric strain increases with strain in all materials. Such strain dependence can be highlighted by observing the corresponding voiding rate. The volumetric strain is found constant for the unfilled EPDM-0, it constantly increases in the case of filled EPDM-40, and is found to increase in filled EPDM, but reaches a saturation value at around λ = 3 before failure (Figure 3b). No strain rate effect of the volumetric strain and on voiding rate is observed in the case of unfilled EPDM-0. However, the strain rate effect yields an increased volumetric strain and voiding rate in filled EPDM-40 and EPDM-80 (Figure 3a). Such a strain rate effect is more visible by increasing the filler content (Figure 3c).

The absence of changes in volumetric strain in unfilled EPDM-0 by increasing the strain rate is consistent with the absence of significant changes of the chains network alteration (Figure 1), confirming the increased stress with strain rate (Figure 2) to be essentially due to isochoric viscoelastic effect, i.e., increased density of trapped entanglements and/or chains friction. Non negligible changes of the volumetric strain in EPDM-40 suggest the effect of strain rate on voiding to be ascribe to damage of the filler network, while the rubber network is relatively preserved (cf. Figure 1).

In filled EPDM-80, making the strong assumption that the volumetric strain is totally ascribed to chains network alteration in the rubbery matrix (no damage associated with the filler network), the relative loss of chains network by increasing the strain rate from 10^−2^ s^−1^ and 1 s^−1^ would have been equal to 30% [35]. This is far from what we observed on the post-swelling test, where variation is found around 1%. Hence, the absence of relation between the damage at chains network scale and DIC damage may be explained by a predominant damage involving the filler network (filler–filler rupture and/or decohesion at filler-rubber interface) in EPDM-80 that accommodates the stress and prevents the chains network alteration. This is consistent with electrical conductivity measurement, showing the effect of the strain rate on filled EPDM to yield a predominant breakdown of the filler–filler network [39]. In addition, the high level of damage (Figure 3a) as well as high cavitation rate developed in that material (Figure 3b) between λ = 2 and λ = 3 may be a cause for the previously observed stress softening prior to failure (Figure 2b,c).

Heat sources have been shown to be a powerful means to detect the occurrence of damage in filled rubber deformed in adiabatic conditions [40]. In the absence of phase transition, thermomechanical coupling describes elastic (entropic elasticity) and inelastic (viscoelasticity, damage) deformation processes. The integration of elastic mechanisms to time over one cycle is zero. Assuming that for uniaxial tests, the heat source distributions are uniform within the specimen surface (linearization of heat losses), the heat equation can be written [20,41]:

(5) $$\rho C\left(\dot{\theta }+\frac{\theta }{\tau }\right)=S$$

where *ρ* is the bulk density (g·cm^−3^) of the EPDM materials, *C* its heat capacity (J·g^−1^·°C^−1^), *θ* the temperature variation above the room temperature, $\dot{\theta }$ the rate of heating (W·m^−3^), and *S* the heat source. For the sake of simplicity, as proposed in Ref. [42], a normalized heat source *s* will be defined in the following as the ratio *s = S/ρC* and expressed in °C·s^−1^. The time constant τ (s) characteristic of the heat loss along the specimen thickness is given by τ=ρCe/2h, where *e* is the thickness of the specimen, and *h* the convection coefficient. The time constant τ_0_ = 27 s of the non-deformed specimen has been calculated in a previous study by rapidly stretching the rubber specimen up to failure to induce self-heating and following the cooling down to room temperature [43]. The dependence of this time constant to the applied deformation was then identified assuming a thickness reduction with longitudinal deformation, yielding to $\tau =\tau_{0}/\sqrt{\lambda_{1}}$. Constant times over the strain range studied were found to be in the range of those obtained by Samaca Martinez et al. on Natural Rubber (NR) and Styrene Butadiene Rubber (SBR) [20,40,42].

As expected, for all studied strain rates, self-heating increases with filler content (Figure 4) due to higher dissipation (viscoelasticity, damage). Measured thermal dissipation becomes predominant with the increase of strain rate because the experiments get closer to adiabatic conditions (Figure 4a–c). This is reflected by an increase of both the temperature and the temperature rate (see Equation (5)), yielding then in an increase of the heat source with strain rate in all materials (Figure 4d,e). For the highest strain rate tested (1 s^−1^), the heat source in unfilled, and filled EPDM measured at a moderate strain (below λ = 2) shows a rapid increase with strain, likely ascribed to the accumulation of the dissipative viscoelastic effects. Indeed, damage estimated by volumetric strain remains weak in this strain range for all EPDM (Figure 3). At larger strain (above λ= 2), the heat source stabilizes and even decreases in unfilled rubber, suggesting no measurable new source of heat dissipation. Contrarily, heat source further increases in filled EPDM-40 and EPDM-80, due to possible dissipative viscosity and/or damage at such high strain. In particular, damage is found to increase notably in this strain range for both filled EPDM-40 and EPDM-80 (Figure 3). Additionally, EPDM-80 subjected to the largest strain rate undergoes a sharp peak of the heat source at λ = 3 (Figure 4f) that we ascribe to damage, as reflected by a high voids fraction (Figure 3b) and the high voiding rate that reaches a saturation value at the same strain λ = 3 (Figure 3c). This high level of dissipative damage may participate in the stress softening occurring at the same strain (Figure 2b).

One may note that damage is identified here by self-heating using a passive thermography technique (without external heat). However, any internal heat source induced by the deformation process may in turn retroactively modify both the viscoelastic and the damage behavior, especially by accumulating mechanical cycles. In these conditions, a modification of the strain rate raises the question of how self-heating intervenes in cyclic loadings, where damage may accumulate or conversely accommodate, governing then the subsequent macroscopic failure.

### 3.3. Effect of the Strain Rate on Damage During Cyclic Loading

**Figure 5:**
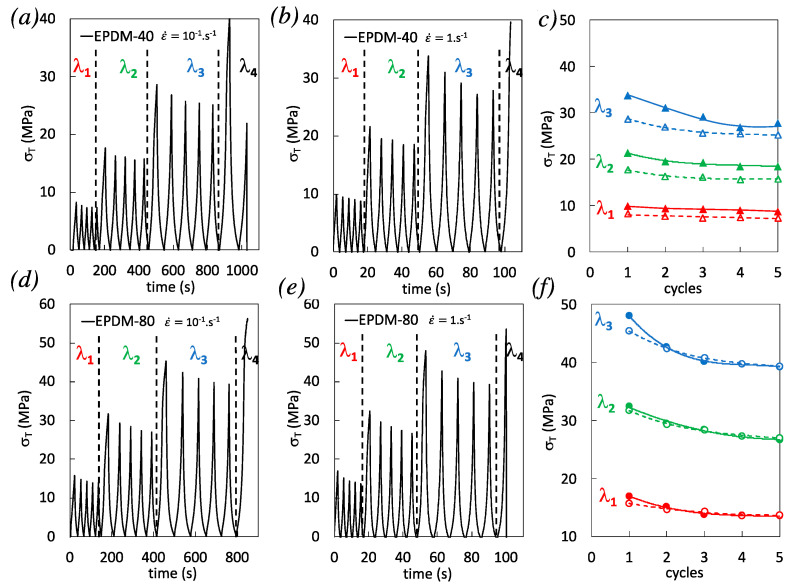
(**a**–**b**) True stress at the strain rates 10^−1^ s^−1^ and 1 s^−1^ for EPDM-40. (**c**) Corresponding maximum stress reached during multiple cyclic loadings performed at three different stretching ratios λ_1_ = 2 (in red), λ_2_ = 2.9 (in green), and λ_3_ = 3.7 (in blue), and for the strain rates 10^−1^ s^−1^ (unfilled symbols) and 1 s^−1^ (filled symbols). (**d**–**e**) True stress at the strain rates 10^−1^ s^−1^ and 1 s^−1^ for EPDM-80. (**f**) Corresponding maximum stress reached during multiple cyclic loadings performed at three different stretching ratios λ_1_ = 2 (in red), λ_2_ = 2.9 (in green), and λ_3_ = 3.7 (in blue), and for the strain rates 10^−1^ s^−1^ (unfilled symbols) and 1 s^−1^ (filled symbols).

We have observed in the previous section that the increase of strain rate during single uniaxial tension of filled EPDM yields at high strain (prior failure) in a significant increase of damage (Figure 3) and self-heating (Figure 4) participating in a stress softening (Figure 2). In this section, we aim to investigate the strain rate effect during cyclic loading. To evaluate the coupling between damage (DIC) and self-heating (IR) under cyclic conditions, the specimens have experienced incremental loadings (Figure 5): 5 cycles with loading up to a fixed strain and unloading down to zero stress, successively up to λ_1_ = 2, λ_2_ = 2.9, λ_3_ = 3.7, and λ_4_ (specimen failure before reaching λ_4_). This procedure is performed up to the failure of the specimen and for two different nominal strain rates, 10^−1^ s^−1^ and 1 s^−1^. First, consistent with what was observed during single loading, the effect of the strain rate on the stress reached during the loading phase is hardly visible with higher filler content EPDM-80 (compare all 1st cycles for both strain rates in Figure 5f), which may partly be ascribed to the predominant damage developed during the loading phases, as we have seen in the previous section. Second, both filled EPDM-40 and EPDM-80 exhibit stress softening by cyclic accumulation (compare cycles from 1 to 5), a typical feature of Mullins effect [44]. Such softening increases by increasing the strain rate (Figure 5c,f). As a result, the stress values from the 1st to the 5th cycles performed at the highest cyclic strain λ_3_ become closer in EPDM-40 (Figure 5c) and become similar in EPDM-80 (Figure 5f) for the two strain rates tested. To further evaluate the dissipative nature of such stress softening accumulated over the cycles, a characterization of the mechanical and thermal dissipation is performed.

The relationship between mechanical dissipation and thermal dissipation during cyclic loading is provided by the calculation of the corresponding dissipated energies. The mechanical energy *W_def_* is split into elastic *We* and anelastic *Wan* parts. The former part is the recoverable elastic energy per unit of volume. The latter part, i.e., the total anelastic energy per unit volume, is made of intrinsic dissipation φ_1_ and stored energy per unit volume *Ws*. In the following, the elastic component per cycle remains close to zero and it can be considered that the mechanical and anelastic energy per unit volume per cycle are approximately the same. The deformation energy is then written:

(6) $$W_{def}=\varphi_{1}+W_{s}$$

The hysteresis loops that characterizes the deformation energy loss is essentially due to material dissipation. In the absence of microstructural transformations, the area of the surface of the hysteresis loop equals the amount of energy dissipated in the material over a complete loading cycle. The mechanical energy per cycle was estimated using the expression below:

(7) $$W_{def}=\int \;\frac{Fd\lambda_{1}}{L_{0}e_{0}\lambda_{2}\lambda_{3}}$$

The heating of the specimen is associated with the dissipated energy. It is often characterized by a progressive increase in temperature variations, as we have seen in the single loading tests (Figure 4). The mean dissipation energy rate over a cycle ~φ_1_ was then calculated via the zero-dimensional approach of the local heat diffusion equation:

(8) $$\varphi_{1}=\rho C\int \;\left(\dot{\theta }+\frac{\theta }{\tau }\right)dt$$

The macroscopic densities of the EPDM, *ρ*, are calculated given their carbon black fraction and the known density of the unfilled EPDM around 0.87 g.cm^−3^. The density of the filled EPDM-40 and EPDM-80 are found equal to 1.16 and 1.31 g.cm^−3^, respectively. The heat capacity of the vulcanized EPDM is assumed to be independent of the temperature in the range of temperature variations induced by self-heating. From the literature [45], the heat capacity is found to be around 2.3 J·g^−1^·°C^−1^ in vulcanized unfilled EPDM. Heat capacity of the filled EPDM were also taken from the literature. It was found to slightly decrease down to 2.24 and 2.06 in EPDM containing N550 carbon black particles [46] (same than in our study), corresponding to a loading of 22 vol.% and 44 vol.% of carbon black content, respectively, matching with the weight fraction of our EPDM-40 and EPDM-80 respectively.

**Figure 6:**
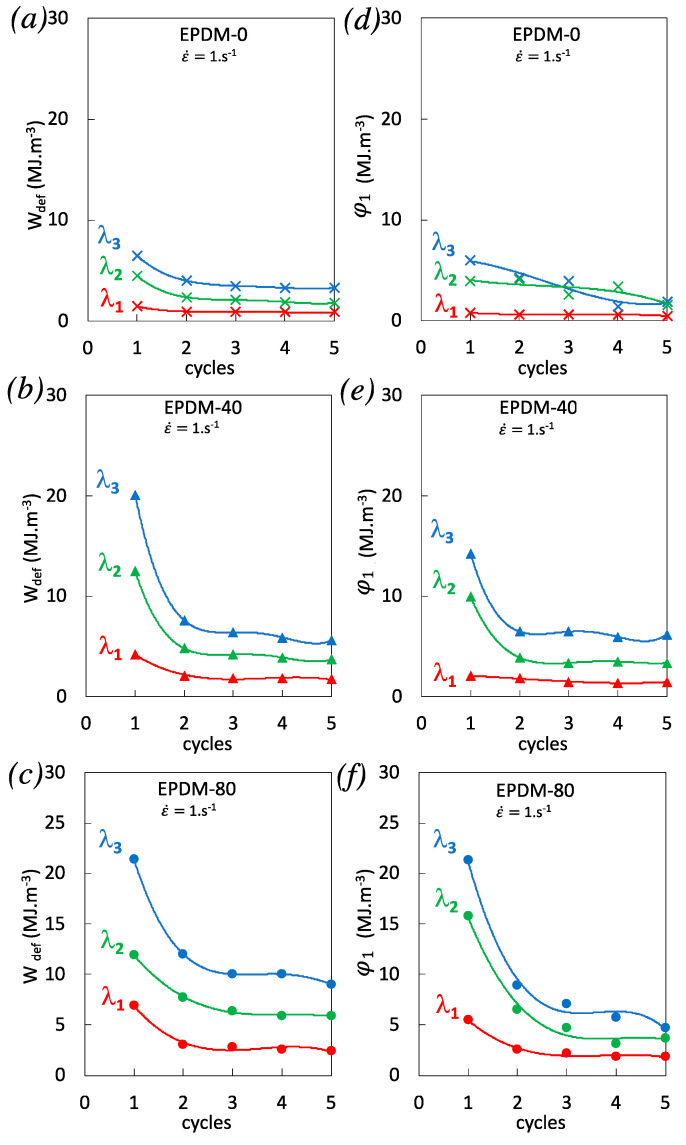
Deformation energy estimated from the area under the stress–strain curve (Equation (7)) for EPDM-0 (**a**), EPDM-40 (**b**), and EPDM-80 (**c**) and dissipated energy estimated from the integration over each cycle of the heat source (Equation (8)) for EPDM-0 (**d**), EPDM-40 (**e**), and EPDM-80 (**f**). The energies are calculated for the three different stretching ratios λ_1_ = 2 (red data points), λ_2_ = 2.9 (green data points), and λ_3_ = 3.7 (blue data points) and for the strain rate 1 s^−1^.

Mechanical energy and thermal energy dissipation are shown in Figure 6 for the highest strain rate tested. where the unfilled EPDM-0 is also added for sake of comparison. It is observed that the thermal dissipation shows more disparities as compared to mechanical energy, which is likely due to the supplementary analytical treatment using a higher number of materials parameters to deduce the thermal energy. Nonetheless, results would suggest that most of the non-reversible mechanical energy is dissipated into heat. Conversely, considering the observed disparities in the estimate of energy dissipation from the heat source, it is difficult to clearly discuss on the contribution of stored energy in the energy of deformation. The dependences of the mechanical and thermal dissipations on the carbon black content and on the cyclic accumulation are relatively similar. In particular, a weak dependence in the unfilled EPDM-0 is observed, while there is a significant decrease from the first to the following cycles in the filled EPDM-40 and EPDM-80, suggesting the largest contribution of dissipative mechanisms to occur during the first cycles, consistent with the Mullins phenomena.

**Figure 7:**
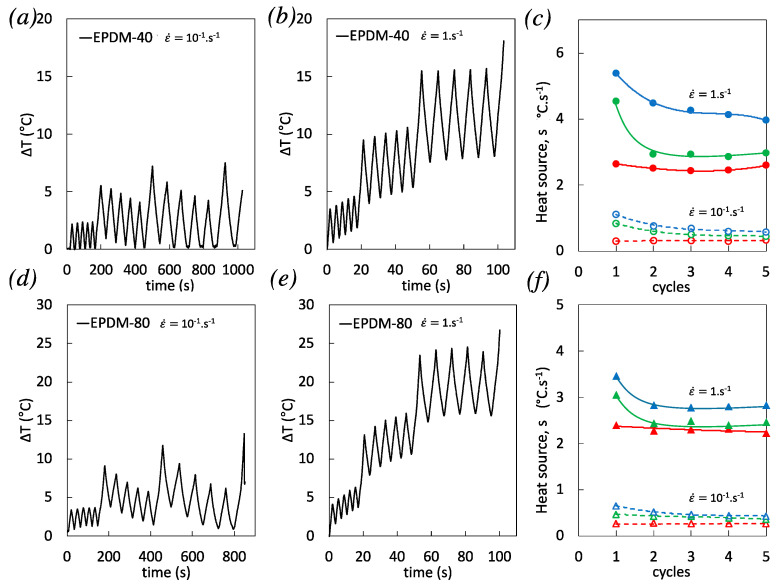
*(***a**–**b***)* Self-heating at the strain rates 10^−1^ s^−1^ and 1 s^−1^ for EPDM-40. *(***c***)* Corresponding temperature rise measured between the minimum and maximum strain reached during the loading phase of the cyclic loadings performed at three different stretching ratios λ_1_ =2 (in red), λ_2_ = 2.9 (in green), and λ_3_ = 3.7 (in blue), and for the strain rates 10^−1^ s^−1^ (unfilled symbols) and 1 s^−1^ (filled symbols). *(***d**–**e***)* Self-heating at the strain rates 10^−1^ s^−1^ and 1 s^−1^ for EPDM-80. *(***f***)* Corresponding temperature rise measured between the minimum and maximum strain reached during the loading phase of the cyclic loadings performed at three different stretching ratios λ_1_ = 2 (in red), λ_2_ = 2.9 (in green), and λ_3_ = 3.9 (in blue), and for the strain rates 10^−1^ s^−1^ (unfilled symbols) and 1 s^−1^ (filled symbols).

Self-heating and heat source measured during the cyclic tests on EPDM-40 and EPDM-80 are now shown for the two strain rates studied, 10^−1^ s^−1^ and 1 s^−1^. Cyclic tests performed at the lowest strain rate generate little self-heating (Figure 7a,d). For this strain rate and for both materials, self-heating amplitude over a cycle is increased by increasing the applied strain, but the average self-heating is found to only weakly vary above room temperature. Contrarily, at the highest strain rate, self-heating accumulates with the cycles sequence in both materials, up to a maximum value of RT + 27 °C in highly filled EPDM-80 prior to failure (Figure 7b,e). Self-heating accumulation during high strain rate cyclic loading is partly due to high viscous dissipation like interfacial sliding (friction) at the filler–rubber interfaces [47,48]. In addition, the breakage (damage) and reformation of inter-aggregate bonds have also been identified as a major heat dissipation during cyclic deformation of filled rubber [49]. The heat source reached at maximum strain during the cyclic tests is found to be predominant for the first cycle of the series, and then seems to stabilize with the accumulation of cycles (Figure 7c,f). Like in the case of single loading, to attest for the role of damage in these dissipative mechanisms, a direct investigation on the opening and closing of cavities is proposed in the following by using Digital Image Correlation (DIC).

Due to high level of self-heating reached during the highest strain rate cycles, its effect on thermal expansion, i.e., on the measure of volumetric strain, had been evaluated in a previous study [43]. It resulted that volume expansion due to self-heating was found negligible as compared to the one induced by damage. Hence, in the following, the volumetric strain measured during cycles is considered to be a suitable measure of the strain induced voiding fraction.

**Figure 8:**
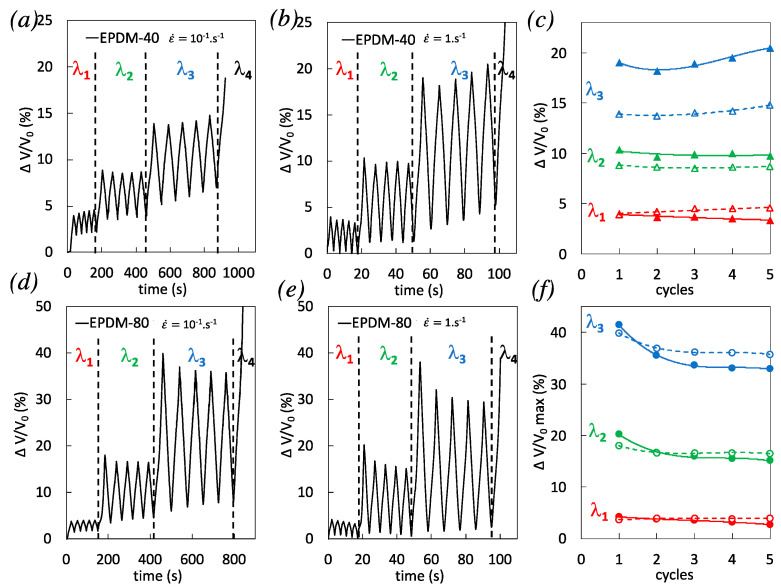
(**a**–**b**) Volumetric strain at the strain rates 10^−1^ s^−1^ and 1 s^−1^ for EPDM-40. (**c**) Corresponding maximum volumetric strain reached during multiple cyclic loadings performed at three different stretching ratios λ_1_ = 2 (in red), λ_2_ = 2.9 (in green), and λ_3_ = 3.7 (in blue), and for the strain rates 10^−1^ s^−1^ (unfilled symbols) and 1 s^−1^ (filled symbols). (**d**–**e**) Volumetric strain at the strain rates 10^−1^ s^−1^ and 1 s^−1^ for EPDM-80. (**f**) Corresponding maximum volumetric strain reached during multiple cyclic loadings performed at three different stretching ratios λ_1_ = 2 (in red), λ_2_ = 2.9 (in green), and λ_3_ = 3.7 (in blue), and for the strain rates 10^−1^ s^−1^ (unfilled symbols) and 1 s^−1^ (filled symbols).

In EPDM-40, the dependence of volumetric strain on cycles accumulation is not obvious. However, a significant decrease from the first cycle to following cycles in EPDM-80, especially for the highest strain rate tested, is concomitant with decreased heat source over cyclic accumulation (Figure 7f). This is in line with the literature showing the major role of cavitation during the first cycle of incremental cyclic loadings [50] and with the participation of filler damage to heat dissipation during cyclic deformation of filled rubber [49]. Consistent with results obtained for single tensile tests (Figure 3), a higher volumetric strain was observed during the first loading phases in EPDM-40 and EPDM-80 when applying the highest strain rates (compare the cycles 1 for both strain rates in Figure 8c,f). While there was no clear effect of strain rate on volumetric strain during cyclic accumulation in EPDM-40 (Figure 8c), the volumetric strain in EPDM-80 deformed with the highest strain rate significantly decreases, and after a series of 5 cycles, it is found lower than for the same material deformed at the lowest strain rate (Figure 8f).

**Figure 9:**
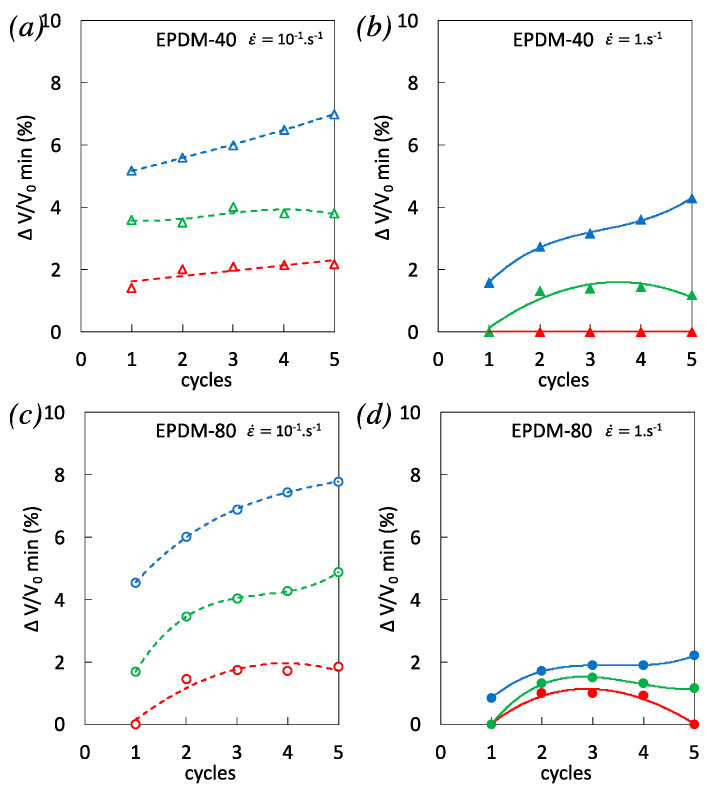
Minimum volumetric strain reached during multiple cyclic loadings performed at three different stretching ratios λ_1_ = 2 (in red), λ_2_ = 2.9 (in green), and λ_3_ = 3.7 (in blue), and for the strain rates 10^−1^ s^−1^ (unfilled symbols) and 1 s^−1^ (filled symbols) for EPDM-40 (**a**–**b**) and EPDM-80 (**c**–**d**).

Another point of interest is the volumetric strain at the end of the unloading phases (Figure 9), which is found to be the lowest for the highest strain rate, for both filled EPDM-40 and EPDM-80. This suggests a better ability of these materials to close cavities upon unloading, during high strain rate cycles. It has been show that no delayed cavitation is expected after rapid loading [27], so that a kinetic involving a delayed cavities nucleation/growth is assumed not to be the main cause of the effect of strain rate on cavities closing upon unloading. In quasi-static conditions (weak self-heating), cavities closing upon unloading had been ascribed to filler–filler re-aggregation or reattachment of the polymer chains to fillers [51]. Such filler network accommodation appears to be favorable to the preservation of the bulk rubber chains network (Figure 1). Additionally, by increasing the strain rate in cyclic loading, the significant rise in temperature (especially in highly filled rubber) may yield a supplementary reduction of the local confinement of the rubber in the filler vicinity [52] that in turn is expected to contribute to limit the development of cavitation. Hence, the combination of filler network accommodation and self-heating in highly filled rubber during the accumulation of high strain rate cycles is a possible cause of (i) the limitation of cavities opening during loading, (ii) favoring of cavities closing during unloading, and (iii) the increased stress softening with cyclic accumulation that may participate to prevent premature failure.

## 4. Conclusions

The effect of the strain rate on damage in carbon black filled EPDM during single and cyclic loading was investigated. This has been performed by analyzing the stress–strain response, the voiding fraction by Digital Image Correlation (DIC), the heat source by Infrared Thermography (IR), and the chains network alteration by swelling experiments.

In single loading conditions, post-swelling performed on mechanically tested specimens suggested the rubber network can be preserved by increasing the strain rate of single loading, while preferential damage occurs in the filler network. Thermomechanical study showed that such damage is associated at high strain rate (1. s^−1^) with a stress softening and an adiabatic heat source rise.

Cyclic loadings were found to favor the preservation of rubber network as compared to single loading. This was explained by a possible re-organization (accommodation) of the filler network within the cycles. If the strain rate of the cyclic loading increases to reach near adiabatic conditions (1 ^−1^), the combination of this filler network accommodation with substantial self-heating in highly filled EPDM is proposed to be a possible cause of the limitation of cavities opening during loading and the facilitation of cavities closing during unloading. The combined effect of filler network accommodation and self-heating with cyclic accumulation is thought to be at the origin of (i) a higher stress softening and (ii) the prevention of premature failure. Hence, the selection of high strain rate and cyclic accumulation to intentionally “damage” the rubber network in industrial applications such as mechanical devulcanization is proposed to be optimized by considering the loading conditions (number of cycles, loading rate, loading sequence), but also by combining it with other sources of devulcanization (biological, chemical, physical devulcanization, etc.) in order to improve the selective breakage of the chemical crosslinks in the rubber network.

## Figures and Tables

**Table 1:** Composition of the EPDM materials.

Components	Content (phr)	*ρ* * (mol·cm^−3^)
EPDM	100	0.87
Carbon black (N550)	0, 40, 80	2.00
Calcium Oxide	4	3.34
Zinc Oxide	5	5.61
Stearin	1	0.94
Polyethylene Glycol (PEG)	2	1.12
Sulfur	1.2	2.00
Mercaptobenzothiazole (MBT 75%)	1	1.46
Mercaptobenzothiazole disulfide (MBTS 75%)	0.8	1.50
N-cyclohexyl-2-benzothiazolesulfenamide (CBS 75%)	1.2	1.30
Zinc dialkyl dithiophosphate (ZDTP 70%)	1.5	1.20

* *ρ* is the density of the different components of the EPDM materials expressed in mol·cm^−3^.
